# Alternative transmission routes in the malaria elimination era: an overview of transfusion-transmitted malaria in the Americas

**DOI:** 10.1186/s12936-017-1726-y

**Published:** 2017-02-15

**Authors:** Regina M. Alho, Kim Vinícius Amaral Machado, Fernando F. A. Val, Nelson A. Fraiji, Marcia A. A. Alexandre, Gisely C. Melo, Judith Recht, André M. Siqueira, Wuelton M. Monteiro, Marcus V. G. Lacerda

**Affiliations:** 1 Universidade do Estado do Amazonas, Av. Pedro Teixeira, 25, Dom Pedro, Manaus, AM 69040-000 Brazil; 2 Fundação de Hematologia e Hemoterapia do Amazonas, Av. Constantino Nery, 4397, Chapada, Manaus, AM 69050-002 Brazil; 3 Fundação de Medicina Tropical Dr. Heitor Vieira Dourado, Av. Pedro Teixeira, 25, Dom Pedro, Manaus, AM 69040-000 Brazil; 4 Recife, PE Brazil; 5 Instituto Nacional de Infectologia Evandro Chagas, Fundação Oswaldo Cruz, Av. Brasil, 4365, Manguinhos, Rio de Janeiro, RJ 21040-360 Brazil; 6 Instituto de Pesquisas Leônidas & Maria Deane, Fundação Oswaldo Cruz, Rua Terezina, 476, Adrianópolis, Manaus, AM 69057-070 Brazil

**Keywords:** Malaria, *Plasmodium*, Blood transfusion, Transfusion-transmitted malaria

## Abstract

**Background:**

Transfusion-transmitted (TT) malaria is an alternative infection route that has gained little attention from authorities, despite representing a life-threatening condition. There has been no systematic review of this health problem in American countries. The aim of this study was to describe the clinical and epidemiological characteristics of TT malaria in the Americas and identify factors associated with lethality based on the studies published in the literature.

**Methods:**

Potentially relevant papers in all languages were retrieved from MEDLINE and LILACS. Additional articles were obtained from reviews and original papers. Publications on screening of candidate blood donors and on surveillance of TT malaria cases were included. Odds ratios with respective 95% confidence intervals (95% CI) were calculated. Epidemiological characteristics of blood donors of TT malaria cases, including a pooled positivity of different tests for malaria diagnosis, were retrieved.

**Results:**

A total of 63 publications regarding TT malaria from seven countries were included, from 1971 to 2016. A total of 422 cases of TT malaria were recorded. Most TT malaria cases were in females (62.0%) and 39.5% were in the ≥61 years-old age group. About half of all cases were from Mexico (50.7%), 40.3% from the United States of America (USA) and 6.6% from Brazil. Gyneco-obstetrical conditions (67.3%), surgical procedures (20.6%) and complications from neoplasias (6.1%) were the most common indications of transfusion. Packed red blood cells (RBCs) (50.7%) and whole blood (43.3%) were the blood products mostly associated with TT malaria. Cases were mostly caused by *Plasmodium malariae* (58.4%), followed by *Plasmodium vivax* (20.7%) and *Plasmodium falciparum* (17.9%). A total of 66.6% of cases were diagnosed by microscopy. Incubation period of 2–3 weeks was the most commonly observed (28.6%). Lethality was seen in 5.3% of cases and was associated with living in non-endemic countries, *P. falciparum* infection and concomitant neoplastic diseases.

**Conclusion:**

There is an important research and knowledge gap regarding the TT malaria burden in Latin American countries where malaria remains endemic. No screening method that is practical, affordable and suitably sensitive is available at blood banks in Latin American countries, where infections with low parasitaemia contribute greatly to transmission. Lethality from TT malaria was not negligible. TT malaria needs to be acknowledged and addressed in areas moving toward elimination.

**Electronic supplementary material:**

The online version of this article (doi:10.1186/s12936-017-1726-y) contains supplementary material, which is available to authorized users.

## Background

Malaria is an infectious disease caused by parasites of the genus *Plasmodium* that is characterized by a potentially fatal acute febrile illness. It is considered a great public health problem worldwide. According to the World Health Organization (WHO), about 3.2 billion people were at risk of developing malaria globally in 2015, with 212 million new cases and 429,000 deaths estimated [1]. Malaria transmission is widespread, with cases concentrated mostly in tropical and sub-tropical areas, mainly in Central and South America and in Southeast Asia for *Plasmodium vivax* [2] and in Africa for *Plasmodium falciparum* [3]. Clinically, malaria can manifest with severe symptoms, which can evolve to lethal cases, or present as asymptomatic infections. The latter are particularly challenging in non-endemic settings and those approaching elimination, as asymptomatic *Plasmodium* parasites in low densities are still able to transmit infection but less likely to be diagnosed.

In the Americas, the population at risk for malaria exceeds 120 million in over 21 countries, with *P. vivax* infections accounting for more than 70% of reported cases [1]. Only five countries in this region have higher than 40% *P. falciparum* infection rate (Haiti, Dominician Republic, Guyana, French Guiana, Suriname). Out of 390,000 confirmed cases in the Americas in 2015, three countries were responsible for more than 75%: Brazil (37%), Venezuela (23%) and Colombia (17%) [1]. In Brazil, malaria transmission is mainly restricted to the Amazon region with 99.8% of all reported cases, of which 83% are *P. vivax* malaria [4]. Despite an 80% decline in mortality over the past 15 years, 79 malaria-related deaths were reported in the Americas [1]. Interestingly, the highest lethality is seen outside the endemic regions, where a higher proportion of *P. falciparum* combined with late diagnosis may lead to higher rates of complications [5].

The natural malaria transmission occurs through the blood meal bite of the female *Anopheles* mosquito, although infection may also occur congenitally or accidentally, through infected blood transfusion. Transfusion-transmitted (TT) malaria is an alternative infection route that has gained little attention from authorities, despite presenting real danger to patients. Subjects requiring blood transfusion are a vulnerable population in debilitating conditions. Vivax malaria was traditionally considered a benign disease with no associated severe cases. Recent data however, have shown that vivax malaria may result in severe disease and death [6–8] including in the Americas [10–14]. The Brazilian Ministry of Health has shown that although the number of hospitalizations and severe cases in Brazil has decreased in recent years, deaths due to malaria infection still occur, with a total of 60 deaths in 2012 and 41 in 2013 [9]. A case series of autopsies in which *P. vivax* was considered a causative agent or a contributing factor for death showed that 13 out of 17 patients presented co-morbidities [15].

Blood banks usually perform a screen of donor candidates’ health before blood donation, including current infections and a detailed history of previous diseases and travel. The criteria for this haemovigilance are defined by each country through their national regulation agencies. In Brazil, policies regarding blood donation differ between malaria endemic and non-endemic regions. In non-endemic areas, candidates will not be accepted as donors if they have been to endemic areas within the previous 6 months or had malaria in the previous 3 years, while in areas with active malaria transmission, the presence of *Plasmodium* or plasmodial antigens must be tested [16, 17]. A recent study in the Amazon region, that evaluated ten blood banks regarding infrastructure and procedures relative to TT malaria, found that none was classified as ‘adequate’, highlighting an urgent need to call attention to this challenge and work towards better standardization and regulation compliance regarding TT malaria by authorities [18]. Furthermore, as most malaria-infected blood donors are asymptomatic, both the use of clinical and epidemiological criteria as well as microscopy and rapid diagnostic tests (RDTs) are potentially insufficient for malaria screening. These asymptomatic infections characterized by low parasite densities require more sensitive methods for detection [19]. Although cases of TT malaria are assumed based on different criteria, transmission can only be proven definitely by genotyping. However, most cases in the literature are “presumed” cases, where genotyping was not done or reported.

The primary objective of this study was to describe epidemiological characteristics of TT malaria in the Americas through a systematic review. Secondary objectives included description of donor epidemiology characteristics, donor selection process in malaria endemic and non-endemic regions, diagnostic methods used in donor screening, and demographical, clinical and laboratory characteristics of TT malaria.

## Methods

A systematic review was conducted of all available articles regarding TT malaria in countries of the Americas. Potentially relevant articles in English, Spanish and Portuguese were accessed from MEDLINE and LILACS to review their full texts. A free text search using the combination of Medical Subject Heading (MeSH) terms and keywords was utilized (Table 1). Only original research was included. Studies were eligible for inclusion if they reported any information on TT malaria in subjects living in American countries. An individual was considered to have malaria if they presented with a positive diagnostic test, such as microscopy, direct serology, RDT, or DNA amplification tests. Moreover, positive indirect serology was considered a marker of lifetime *Plasmodium* infection [4]. After eliminating duplicates, relevant papers were identified by screening titles and abstracts. Two independent investigators performed the selection, with disagreements being included after consensus was reached. Citation tracking of reviews and original papers resulted in inclusion of additional articles. Data were extracted by filling out a template with study area, study design, participant characteristics, number of cases, diagnostic methods, and *Plasmodium* species. Afterwards, articles were classified in the following categories:

**Table 1:** Keywords and MESH headings used for literature searches

Database	Search strategy
LILACS	(Transfusion) AND (malaria OR plasmodium)
Medline	(Transfusion) AND (malaria OR plasmodium) AND (Antilles OR Latin America OR South America OR Central America OR Caribbean OR Anguilla OR Antigua OR Aruba OR Barbuda OR Argentina OR Bahamas OR Barbados OR Belize OR Bolivia OR Brazil OR Chile OR Colombia OR Costa Rica OR Dominica OR Dominican Republic OR Ecuador OR El Salvador OR Grenada OR Grenadines OR Guadeloupe OR Guatemala OR Guyana OR Haiti OR Honduras OR Jamaica OR Martinique OR Mexico OR Montserrat OR Nevis OR Nicaragua OR Panama OR Paraguay OR Peru OR Puerto Rico OR Saint Kitts OR Saint Lucia OR Saint Vincent OR Suriname OR Surinam OR Trinidad OR Tobago OR Uruguay OR Venezuela OR North America OR USA OR Canada)

### Epidemiological screening of candidate blood donors

Cross-sectional information from donations deferral based on epidemiological history was collected, including country and city of the study, time period, subjects characteristics, sample size, and deferral causes and rates.

### Laboratorial screening of candidate blood donors

*Plasmodium* infection prevalences were collected from different study areas, including sample size and characteristics, laboratory method employed and prevalence rates by species.

### Case reports of TT malaria

In this category, data extraction was performed at individual level to identify the profile of TT malaria cases, including country of the report, gender, age, transfusion indication, blood product associated with TT malaria, *Plasmodium* species, method employed for malaria diagnosis, incubation period, and signs and symptoms presented by the patient.

Proportions of deaths from the pooled TT malaria cases were compared by Chi square test (corrected by Fisher’s test if needed), using living in an endemic country, gender, age group, transfusion indication, blood product associated with TT malaria, *Plasmodium* species, and incubation period as independent variables. Odds ratio (OR) with its 95% confidence interval (95% CI) was calculated for each association. Where zeros caused problems with computation of the OR and 95% CI, 0.5 was added to all cells [20, 21]. Statistical analysis was performed using STATA package version 14.1 (Stata Corp. 2013).

Epidemiological characteristics of blood donors of TT malaria cases were retrieved, including gender, age, history of living or travelling to endemic areas, probable site of infection, history of previous episodes of malaria, *Plasmodium* species found and detection methods evidencing *Plasmodium* infection. Positivities of different tests for malaria diagnosis in blood donors were presented.

## Results

### Study characteristics

**Fig. 1:**
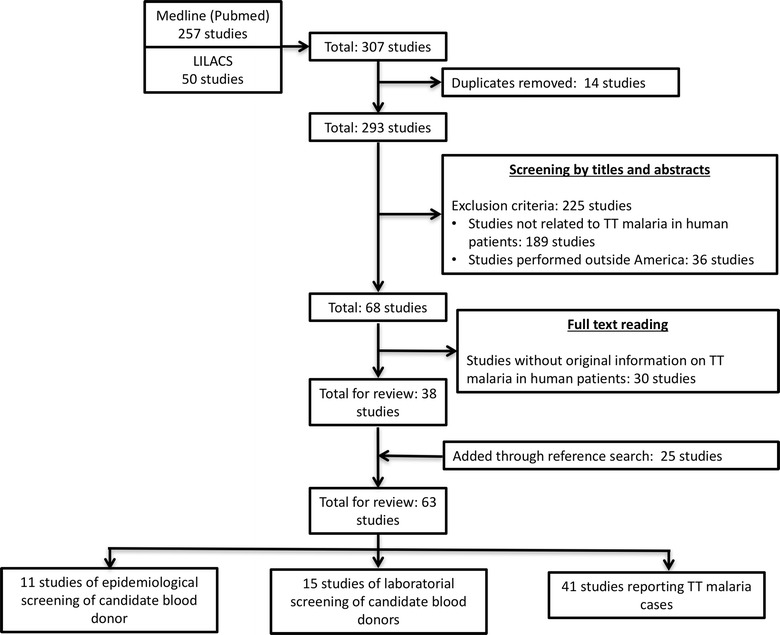
Flow chart of inclusion. Four articles presented more than one type of study category

An initial database search returned 257 records from MEDLINE and 50 from LILACS. After duplicate removal, 293 studies were assessed for inclusion criteria compliance. Screening by titles and abstracts revealed 68 potential inclusions. After full text reading, 30 articles were removed. Finally, 25 additional records were identified through manual search of reference lists. A total of 63 records referred to TT malaria in the Americas and were retrieved for inclusion (Fig. 1). From these, 53% (34) were from the United States of America (USA), 18% (11) from Brazil, 8% (five) from Canada, 8% (five) from Venezuela, 6% (four) from Colombia, 5% (three) from Mexico, and 2% (one) from Peru. A total of 11 studies of epidemiological screening of candidate blood donors, 15 studies of laboratorial screening of candidate blood donors and 41 studies reporting TT malaria cases were retrieved.

### Candidate blood donor malaria-related deferrals

Donor selection based on clinical and epidemiological profile was described in 11 records, with five from the USA, three from Canada, two from Brazil, and one from Colombia [22–32] (Table 2). In general, travel or residence in malaria-endemic countries, irrespective of time, were considered as deferral causes in non-endemic countries. In the USA, data indicated a national deferral rate of 1.1% due to presumed malaria exposure of among 28,933,936 candidate blood donors, from 2000 to 2006 [24], although rates varied between States. In 2006, travel to malaria-endemic countries was responsible for 16.2% of deferrals of blood donors in six blood centres located in San Francisco, Milwaukee, Cincinnati, Pittsburgh, Dedham, and Douglasville [30]. Of this group, 42% were deferred due to travel to low-risk malaria regions in Mexico [31]. In Canada, 7216 donations were deferred due to travel to malaria-endemic countries in 2002 [28]. Nationwide data from Canada estimated a deferral rate of 3% among candidate donors from July to December 2004 [25] and of 4.7% among candidate donors from 2007 to 2012 [26].

**Table 2:** Deferral rates related to malaria exposure in the American continent

Country	Location and period	Period	Subjects	Sample size	Deferral cause	Findings	Reference
Canada	Nationwide data, except Québec	2007–2012	Candidate blood donors	1,625,432	Travel to endemic countries	76,312 deferred subjects	O’Brien et al. [26]
USA	Six blood centres located in San Francisco (CA), Milwaukee (WI), Cincinnati (OH), Pittsburgh (PA), Dedham (MA) and Douglasville (GA)	2006	Overall deferred donors due to travel to endemic countries	2108	Travel to Mexico	885 deferred donors	Spencer et al. [31]
USA	Six blood centres located in San Francisco (CA), Milwaukee (WI), Cincinnati (OH), Pittsburgh (PA), Dedham (MA) and Douglasville (GA)	2006	Overall non-eligible donors at moment	13,007	Travel to endemic countries	2108 deferred subjects	Spencer et al. [30]
Canada	Nationwide data	Jul to Dec 2004	Candidate blood donors	37,165	Travel to endemic countries	1105 deferred subjects	O’Brien et al. [25]
USA	Nationwide data	2000–2006	Candidate blood donors	28,933,936	Travel to endemic countries	316,495 deferred subjects	Leiby et al. [24]
USA	Mississippi Valley Regional Blood Center	Apr 2004 to Mar 2005	Candidate blood donors	NA	Travel or residence in endemic countries	156 deferred subjects	Katz et al. [23]
USA	Six blood centres located in San Francisco (CA), Milwaukee (WI), Cincinnati (OH), Pittsburgh (PA), Dedham (MA) and Douglasville (GA)	2003	Candidate blood donors	NA	Travel to endemic countries	12,310 deferred subjects	Spencer et al. [29]
Brazil	Manaus	NA	Candidate blood donors	324	Previous history of malaria	38 deferrals	Torres et al. [32]
Colombia	Cali	2002	Candidate blood donors	286	Travel or living in endemic areas in the last year	115 deferrals	Castillo et al. [22]
Canada	Nationwide data	2002	Candidate blood donors	NA	Travel to endemic countries	7216 deferred subjects	Shehata et al. [28]
Brazil	São Paulo (SP)	NA	Candidate blood donors	1200	Travel to endemic areas without prophylaxis in the last 6 months, living in malaria endemic areas (deferral for 3 years), previous malaria in the lifetime	36 deferred subjects	Sáez-Alquézar et al. [27]
Brazil	Belém (PA)	NA	Candidate blood donors	250	Travel to endemic area in the last 6 months, previous malaria in the last year or fever in the last 30 days	12 deferred subjects	Sáez-Alquézar et al. [27]
Brazil	Matupá and Peixoto de Azevedo (MT)	NA	Candidate blood donors	31	Fever in the last 30 days	No deferrals	Sáez-Alquézar et al. [27]

*NA* non-available information

Malaria deferral practices were not consistent among endemic countries (Table 2). In Brazil, previous history of malaria, travel to endemic areas without prophylaxis in the last 6 months, living in malaria-endemic areas, travel to endemic area in the last 6 months, and previous malaria in the last year or fever in the last 30 days were considered as deferral causes, depending on the location where the candidate was tested [27, 32]. In São Paulo, a non-endemic area, subjects with history of travel to endemic areas without prophylaxis in the last 6 months, living in malaria-endemic areas (deferral for 3 years) and previous malaria were considered non-eligible for blood donations, resulting in a 3% deferral rate [27]. In malaria-endemic areas of the Brazilian Amazon, deferral donation rates ranged from no deferrals among 31 candidates in the State of Mato Grosso, using fever in the last 30 days as the deferral reason in a hyperendemic area [27], to 11.7% in Manaus where all candidates reporting a previous episode of malaria were deferred [32]. In one report from Colombia, travel or living in endemic areas in the last year was considered as a deferral cause, resulting in a deferral rate in Cali of 40.2% (115) in 2002 in a group of 286 candidate donors [22].

### *Plasmodium* screening of candidate blood donors

Laboratory testing of blood donors (12,370 subjects), deferred donors (5696 subjects) or blood donors suspected to be responsible for TT malaria cases (47 subjects) was shown in 15 studies from Brazil (nine locations), USA (two locations), Venezuela (six locations), and Colombia (two locations) (Table 3). Among selected blood donors, microscopy was used for screening in seven locations, all with negative results. RDT was used in only one study, with no infections detected [33]. PCR was used for donor screening in nine locations, with results ranging from 0.0% in Cali (Colombia) [22] and USA donors [34] to 7.5% in São Paulo [35]. Compared to microscopy, PCR presented higher positivity in one study [32] and total agreement in another [36]. Quantitative buffy coat was employed in two studies with no cases detected [27, 32]. Direct serological tests presented prevalences of 0.0% in São Paulo, Pará and Mato Grosso states (Brazil) [27] and Cali (Colombia) [22] and 2.4% in Venezuela [36]. In endemic countries, indirect serological tests showed prevalences ranging from 0% in hypoendemic areas of Colombia [22] and Venezuela [37] to 64.5% among blood donors of Mato Grosso (Brazil) [27]. In general, serological screening using indirect methods showed circulating anti-*Plasmodium* antibodies with prevalences around 1–2% in non-endemic or hypo-endemic areas of Latin America [27, 37–39] and higher than 10% in malaria-endemic areas [27, 40, 41]. High prevalences of both *P. falciparum* and *P. vivax* antibodies in endemic areas were found with these methods [27, 41].

**Table 3:** Malaria prevalence in candidate blood donors in the American continent

Study area	Sample size and features	Detection method	Prevalence (%)	Prevalence by species (%)	Reference
Manaus, Brazil	407 blood donors	Microscopy	0.0	–	Torres et al. [33]
		RDT	0.0	–	
São Paulo, Brazil	1108 blood donors	PCR	7.5	*P. falciparum* (5.1)	Maselli et al. [35]
				*P. vivax* (2.3)	
				Mixed *P. falciparum*–*P. vivax* infection (0.1)	
USA	5610 malaria-deferred donors	EIA	1.6	NA	Nguyen et al. [34]
		PCR	0.0	–	
Pará, Brazil	595 blood donors	PCR	1.3	*P. vivax* (1.3)	Batista-dos-Santos et al. [42]
Caracas, Ciudad Bolívar, Puerto Ayacucho and Cumaná, Venezuela	762 blood donors	Direct EIA	2.4	NA	Contreras et al. [36]
		Microscopy	0.0	–	
		PCR	0.0	–	
Porto Velho (RO), Brazil	100 blood donors	PCR	3.0	Mixed *P. falciparum*–*P. vivax* infection (2.3)	Fugikaha et al. [43]
Macapá (AP), Brazil	100 blood donors	PCR	3.0	Mixed *P. falciparum*–*P. vivax* infection (2.3)	
Belém (PA), Brazil	100 blood donors	PCR	2.0	Mixed *P. falciparum*–*P. vivax* infection (2.3)	
Rio Branco (AC), Brazil	100 blood donors	PCR	1.0	Mixed *P. falciparum*–*P. vivax* infection (2.3)	
Manaus (AM), Brazil	286 blood donors	Microscopy	0.0	–	Torres et al. [32]
		QBC	0.0	–	
		PCR	0.3	*P. vivax* (0.3)	
Manaus (AM), Brazil	38 deferred donations	Microscopy	0.0	–	Torres et al. [32]
		QBC	1.4	NA	
		PCR	0.0	–	
Caracas, Venezuela	500 blood donors	IIF (IgG Pf)	0.8	*P. falciparum* (0.8)	Contreras et al. [39]
		ELISA (IgG Pf)	0.8	*P. falciparum* (0.8)	
Ciudad Bolívar, Venezuela	500 blood donors	IIF (IgG Pf)	3.8	*P. falciparum* (3.8)	Contreras et al. [39]
		ELISA (IgG Pf)	2.0	*P. falciparum* (2.0)	
Cali, Colombia	286 blood donors	Microscopy	0.0	–	Castillo and Ramírez [22]
		EIA IgG	0.0	–	
		EIA HRPII	0.0	–	
		PCR	0.0	–	
São Paulo (SP), Brazil	1164 blood donors	Microscopy	0.0	–	Sáez-Alquézar et al. [27]
		DIF	0.0	–	
		IIF IgG anti-Pv	–	*P. vivax* (1.2)	
		IIF IgG anti-Pf	–	*P. falciparum* (0.9)	
		ELISA IgG Pf	–	*P. falciparum* (1.0)	
	36 deferred donations	Microscopy	0.0	–	
		DIF	0.0	–	
		IIF IgG anti-Pv	–	*P. vivax* (16.7)	
		IIF IgG anti-Pf	–	*P. falciparum* (5.6)	
		ELISA IgG Pf	–	*P. falciparum* (2.7)	
Belém (PA), Brazil	238 blood donors	Microscopy	0.0	–	Sáez-Alquézar et al. [27]
		QBC	0.0	–	
		DIF	0.0	–	
		IIF IgG anti-Pv	–	*P. vivax* (9.6)	
		IIF IgG anti-Pf	–	*P. falciparum* (10.1)	
		ELISA IgG Pf		*P. falciparum* (17.2)	
	12 deferred donations	Microscopy	0.0	–	
		QBC	0.0	–	
		DIF	0.0	–	
		IIF IgG anti-Pv	–	*P. vivax* (25.0)	
		IIF IgG anti-Pf	–	*P. falciparum* (41.6)	
		ELISA IgG Pf		*P. falciparum* (25.0)	
Matupá and Peixoto de Azevedo (MT), Brazil	31 blood donors	Microscopy	0.0	–	
		DIF	0.0	–	
		IIF IgG anti-Pv	–	*P. vivax* (74.2)	
		IIF IgG anti-Pf	–	*P. falciparum* (64.5)	
		ELISA IgG Pf	–	*P. falciparum* (54.8)	
Porto Velho (RO), Brazil	230 blood donors	IIF IgG Pf	–	*P. falciparum* (32.0)	Ferreira et al. [41]
		IIF IgG Pv	–	*P. vivax* (24.0)	
		IIF IgM Pf	–	*P. falciparum* (4.0)	
		IIF IgM Pv	–	*P. vivax* (1.0)	
Caracas (DF), Venezuela	426 blood donors	EIA IgG Pf	–	*P. falciparum* (0.0)	Nunez et al. [37]
Cumaná (Sucre), Venezuela	120 blood donors	EIA IgG Pf	–	*P. falciparum* (2.5)	Nunez et al. [37]
Barcelona (Anzoátegui), Venezuela	285 blood donors	EIA IgG Pf	–	*P. falciparum* (0.7)	Nunez et al. [37]
San Fernando (Apure), Venezuela	59 blood donors	EIA IgG Pf	–	*P. falciparum* (1.7)	Nunez et al. [37]
Cali, Colombia	1859 blood donors	EIA IgG Pf	–	*P. falciparum* (13.4)	de Morales et al. [40]
Bogotá, Colombia	3114 blood donors	EIA IgG Pf	–	*P. falciparum* (0.9)	de Morales and Espinal [38]
USA	47 (from 117) blood donors suspected to be responsible for 10 TTM cases	IIF Ig anti-Pf+Pv+Pm	19.1	*P. falciparum* (12.8)	Sulzer and Wilson [44]
				*P. malariae* (6.3)	
				*P. vivax* (0.0)	

*DIF* direct immunofluorescence, *EIA* enzyme immunoassay, *IIF* indirect immunofluorescence, *NA* non-available information, *PCR* polymerase chain reaction, *QBC* quantitative buffy coat, *RDT* rapid diagnostic test, *USA* United States of America

In Brazil, reports using PCR showed *P. falciparum* infections in 5.1% of São Paulo blood donors [35], whereas *P. vivax* was found in 2.3% of blood donors in São Paulo [35], 1.3% of blood donors in Belém [42], and 0.3% of blood donors in Manaus [32]; mixed *P. falciparum*–*P. vivax* infections were found in five locations [35, 43]. Among malaria-deferred blood donations, microscopy and PCR tested negative for all samples in the USA [34] and Brazil [32]. Quantitative buffy coat was positive in a sample from Manaus for which PCR was negative [32]. Direct serological tests were negative in São Paulo and Pará states (Brazil) [27]. Indirect serological tests showed a prevalence of 1.6% among 5610 malaria-deferred donors in the USA [34].

In the USA, blood donors suspected to be sources for TT malaria cases tested positive for *P. falciparum* (12.8%) and *P. malariae* (6.3%) using indirect serology [44].

### Epidemiological and clinical profile of TT malaria patients

This systematic review retrieved 422 cases of TT malaria (Table 4). Cases were mostly reported from Mexico (214 cases; 50.7%), the USA (170 cases; 40.3%) and Brazil (28 cases; 6.6%). Most cases were female (62.0%) or belonged to the group age ≥61 years old (39.5%). Gyneco-obstetrical conditions (67.3%), surgical procedures (20.6%) and complications from neoplasias (6.1%) were the most common indications of transfusion. Packed red blood cells (RBCs) (50.7%), whole blood (43.3%) and platelets (6.0%) were the blood products associated with TT malaria. Cases were mostly caused by *P. malariae* (58.4%), *P. vivax* (20.7%) and *P. falciparum* (17.9%). A total of 66.6% of the cases were diagnosed by microscopy and 30.6% by microscopy plus indirect immunofluorescence (IIF). The incubation period was reported in only 10% of all cases, from which 2–3 weeks was the most frequent (28.6%), with 61.9% of cases showing an incubation period of 2–5 weeks.

**Table 4:** Epidemiological characteristics of 422 transfusion-transmitted malaria cases reported in the American continent

Characteristics	Number of cases	Proportion (%)
Country (n = 422; completeness = 100.0%)		
Mexico	214	50.7
USA	170	40.3
Brazil	28	6.6
Canada	4	0.9
Venezuela	3	0.7
Peru	2	0.5
Colombia	1	0.3
Gender (n = 355; completeness = 84.1%)		
Female	220	62.0
Male	135	38.0
Age groups (years) (n = 43; completeness = 10.2%)		
≤5	2	4.7
6–15	2	4.7
16–30	8	18.6
31–45	4	9.3
46–60	10	23.2
≥61	17	39.5
Transfusion indication (n = 165; completeness = 39.1%)		
Gyneco-obstetrical conditions	111	67.3
Surgical procedures	34	20.6
Complications from neoplasias	10	6.1
Haemoglobinopathies	2	1.2
Nephropathy-associated anaemia	2	1.2
Other causes of anaemia	6	4.4
Blood product associated with TTM (n = 150; completeness = 35.5%)		
Packed RBCs	76	50.7
Whole blood	65	43.3
Platelets	9	6.0
*Plasmodium* species (n = 392; completeness = 92.9%)		
*Plasmodium malariae*	229	58.4
*Plasmodium vivax*	81	20.7
*Plasmodium falciparum*	70	17.9
*Plasmodium ovale*	11	2.8
Mixed *P. falciparum*–*P. vivax*	1	0.2
Method(s) employed for malaria diagnosis (n = 314; completeness = 74.4%)		
Microscopy	209	66.6
Microscopy plus IIF	96	30.6
Microscopy plus PCR	5	1.6
Microscopy plus bone marrow aspirate examination	2	0.6
Microscopy plus PCR plus IIF	1	0.3
PCR	1	0.3
Incubation period (in weeks) (n = 42; completeness = 10.0%)		
≤2	3	7.1
2–3	12	28.6
3–4	6	14.3
4–5	8	19.0
5–6	1	2.4
6–7	2	4.8
7–8	7	16.7
>8	3	7.1

*RBCs* red blood cells, *IIF* indirect immunofluorescence, *PCR* polymerase chain reaction

Fever (99.3%) and chills (69.2%) were the most common signs of malaria presented by the TT malaria cases. Signs and symptoms of severe malaria, such as haemoglobin drop, jaundice, convulsions, hypotension, renal failure, respiratory failure, and disseminated intravascular coagulation were also reported. Lethality was 5.3%.

### Risk factors for lethality among TT malaria patients

Living in an endemic country was associated with a lower odd of lethality from TT malaria [OR 0.19 (95% CI 0.06–0.54); (p = 0.002)]. Regarding background conditions, neoplasias were associated with a higher odd of lethality from TT malaria in comparison with other transfusion indications [OR 0.02 (95% CI <0.01–0.51); (p = 0.018)]. *Plasmodium falciparum* malaria was associated with a higher risk of lethality in comparison with *P. malariae* [OR 6.72 (95% CI 2.36–19.13); (p < 0.001)] and *P. vivax* [OR 7.26 (95% CI 1.49–35.41); (p = 0.014)]. Gender, age, blood product received and incubation period were not associated with lethality from TT malaria (Table 5).

**Table 5:** Factors related to death from transfusion-transmitted malaria in the American continent

Variable	Death (number, %)	Discharge (number, %)	OR (95% CI)	p
Living in an endemic country				
Yes	5 (27.8)	215 (67.4)	–	1
No	13 (72.2)	104 (32.6)	0.19 (0.06–0.54)	0.002
Sex				
Male	5 (83.3)	16 (43.2)	–	1
Female	1 (16.7)	21 (56.8)	6.56 (0.70–61.86)	0.100
Age group (in years)				
>60	2 (50.0)	8 (21.6)	–	1
31–60	2 (50.0)	6 (16.2)	0.75 (0.068–6.96	0.800
16–30	0 (0.0)	7 (18.9)	4.41 (0.18–107.29)	0.362
≤15	0 (0.0)	16 (43.2)	9.71 (0.42–225.85)	0.157
Transfusion indication				
Gyneco-obstetrical conditions	0 (0.0)	34 (49.3)	–	1
Surgical procedures	5 (62.5)	24 (34.8)	0.06 (0.01–1.22)	0.068
Complications from neoplasias	2 (25.0)	3 (4.3)	0.02 (<0.01–0.51)	0.018
Haemoglobinopathies	0 (0.0)	2 (2.9)	0.07 (<0.01–4.48)	0.212
Nephropathy-associated anaemia	0 (0.0)	2 (2.9)	0.07 (<0.01–4.48)	0.212
Other causes of anaemia	1 (12.5)	4 (5.8)	0.04 (<0.01–1.24)	0.066
Blood product associated with TT malaria				
Packed RBCs	7 (87.5)	31 (86.1)	–	1
Whole blood	1 (12.5)	4 (11.1)	0.90 (0.09–9.37)	0.932
Platelets	0 (0.0)	1 (2.8)	0.71 (0.03–19.33)	0.842
*Plasmodium* species				
*Plasmodium falciparum*	9 (50.0)	39 (12.4)	–	1
*Plasmodium malariae*	7 (38.9)	204 (65.0)	6.72 (2.36–19.13)	<0.001
*Plasmodium vivax*	2 (11.1)	63 (20.1)	7.26 (1.49–35.41)	0.014
*Plasmodium ovale*	0 (0.0)	7 (2.2)	3.61 (0.19–68.86)	0.394
Mixed *P. falciparum*–*P. vivax*	0 (0.0)	1 (0.3)	0.72 (0.03–19.14)	0.845
Incubation period (weeks)				
≤4	16 (89.9)	75 (69.4)	–	1
>4	2 (11.1)	33 (30.6)	3.52 (0.77–16.19)	0.106

### Characteristics of donors identified as responsible for TT malaria cases

Epidemiological characteristics of 163 blood donors identified as responsible for TT malaria cases are presented in Table 6. Completeness of individual information ranged from 10.4% for age to 82.2% for method evidencing *Plasmodium* infection. Blood donors were mostly male (81.6%) and between 21 and 30 years old (52.9%). History of living or travelling to endemic areas was reported by 96.8% of the donors. Probable site of infection was reported mostly as Sub-Saharan Africa (61.0%), including Nigeria (18.6%), Ghana (10.2%) and Liberia (10.2%). Latin American countries were identified as the probable site of infection for 15.3% of the donors: Central American countries (5.1%), the Brazilian Atlantic coast (3.4%) and Mexico (3.4%). Mediterranean countries (11.9%), Vietnam (8.5%) and India (3.4%) were also identified as probable sites of infection. History of previous episodes of malaria was reported for 33.3% of the donors. *Plasmodium* species found in donors were mostly *P. falciparum* (43.1%), *P. malariae* (27.7%) and *P. vivax* (23.1%). A total of 49.3% of donors were diagnosed by IIF alone and 33.6% by microscopy plus IIF.

**Table 6:** Epidemiological characteristics of 163 blood donors identified as responsible for transfusion-transmitted malaria in the American continent, from 1991 to 2015

Characteristics	Number of cases	Proportion (%)
Sex (n = 103; completeness = 63.2%)		
Male	84	81.6
Female	19	18.4
Age groups (years) (n = 17; completeness = 10.4%)		
17–20	6	35.3
21–30	9	52.9
≥31	2	11.8
History of living or traveling to endemic areas (n = 63; completeness = 38.7%)		
Yes	61	96.8
No	2	3.2
Probable site of infection (n = 59; completeness = 36.2%)		
Sub-Saharan Africa	36	61.0
Nigeria	11	18.6
Ghana	6	10.2
Liberia	6	10.2
Other Sub-Saharan countries	13	22.0
Latin America	9	15.3
Central American countries	3	5.1
Brazilian Atlantic Coast	2	3.4
Mexico	2	3.4
Colombia	1	1.7
Venezuela	1	1.7
Vietnam	5	8.5
India	2	3.4
Mediterranean countries	7	11.9
Greece	4	6.8
Other Mediterranean countries	3	5.1
History of previous episodes of malaria (n = 33; completeness = 20.2%)		
Yes	11	33.3
No	22	66.7
*Plasmodium* species found (n = 130; completeness = 79.8%)		
*Plasmodium falciparum*	56	43.1
*Plasmodium malariae*	36	27.7
*Plasmodium vivax*	30	23.1
*Plasmodium ovale*	7	5.4
Mixed *P. falciparum*–*P. vivax*	1	0.7
Method(s) evidencing *Plasmodium* infection (n = 134; completeness = 82.2%)		
IIF alone	66	49.3
Microscopy plus IIF	45	33.6
Microscopy alone	11	8.2
IIF plus PCR	5	3.7
Microscopy plus IIF plus PCR	3	2.2
PCR alone	3	2.2
Bone marrow aspirate examination alone	1	0.8

Positivities of PCR, microscopy and IIF for malaria diagnosis in blood donors were 73.3, 79.7 and 100.0%, respectively. PCR positivity ranged from 62.5% for *P. falciparum* to 100.0% for *P. vivax.* Microscopy positivity ranged from 66.7% for *P. ovale* to 90.0% for *P. vivax.*

## Discussion

International policies recommend that blood for transfusion should be screened for TT infections, including malaria [45], however these practices vary considerably between countries and regions. Furthermore, there has been a paucity of information concerning the distribution and potential role of different *Plasmodium* species in transfusion-related malaria cases, and the clinical impact of parasitaemic blood amongst patients with co-morbidities, which most of blood transfusion recipients present [19]. This analysis shows that malaria screening in blood services in the American continent is mainly based on the epidemiological history of the donor. Recent travel or residence in malaria-endemic countries was considered as deferral causes in the USA and Canada. In areas with low odds of donors carrying malaria parasites, exclusion of malaria based on history of recent travel or residence in malaria-endemic countries [24, 26] seems a reasonable approach, as it would not greatly impact the amount of blood available for transfusion. In contrast, in some Latin American areas, rejection of malaria-positive donors would jeopardize the blood supply.

In Latin American countries, there is a knowledge gap regarding malaria-deferral practices. In Brazil, previous history of malaria or fever, and travel to or living in endemic areas (with variable time frames), are used as deferral causes. However, evidence-based information is required to guide malaria screening in these areas, especially in those with active malaria transmission, in order to prevent TT malaria and warrant blood availability for transfusion. The wide variation in malaria prevalence in candidate donors from dissimilar endemic areas means that epidemiological and laboratorial screening policies need to be adapted locally. In Brazil, epidemiological screening is based on annual parasite index (API), an indicator not uniform throughout a spatial unit and with considerable changes over time [19]. Strikingly, this screening strategy does not consider that asymptomatic *P. vivax* or *P. ovale* relapses may occur even several months after the primary episode [46]. Cryptic *P. malariae* and *P. falciparum* infections [47–50] are also clearly related to TT malaria in non-endemic areas from asymptomatic individuals returning years after visiting or living in active transmission settings, as shown here for the Americas and previously in a review of TT *P. falciparum* infections in non-endemic countries in Europe and the Americas (Canada and the USA) [51]. In Latin America, malaria is becoming a disease of peri-urban areas, potentially leading to more donations from *Plasmodium* carriers [19].

A major concern in TT malaria epidemiology is to estimate the real burden of *Plasmodium* infection among candidate donors using sensitive detection techniques. Currently there is no available assay to screen blood with low parasitaemia that is sensitive, practical and affordable enough for use by transfusion services [52]. Microscopy is time consuming and certainly not the best diagnostic tool for asymptomatic donors due to its low sensitivity. Rapid diagnostic tests, although faster and reliable, do not offer a substantial improvement in sensitivity compared to microscopy [33].

PCR in donor candidates has shown results ranging from 0% in Manaus [33] to an unexpected frequency of 7.5% in São Paulo [35]. The latter finding shall be taken with caution since it is in disagreement with other publications and possibly overestimates the actual risk of malaria transmission by blood transfusion in the city of São Paulo [53]. Pooled analysis showed that PCR did not substantially increase malaria diagnosis in the identified blood donors compared to microscopy. Only one study suggested PCR as a more sensitive tool to detect low parasitaemias in blood donors in the Amazon compared to microscopy [32]. Newer techniques such as nucleic acid testing (NAT) and loop-mediated isothermal amplification (LAMP) would be the best options today in blood banks [54]. Recently, increased PCR diagnostic sensitivity was achieved by targeting multi-copy genomic sequences [55] and by increasing the volume of blood for DNA extraction [56]. Serological markers remain positive for many years after an initial infection, presenting low specificity in endemic areas. Indeed, indirect serological tests showed high prevalences of *P. falciparum* and *P. vivax* antibodies in malaria-endemic areas, indicating that they are not a good tool for screening donors in those areas [27, 41]. New epitopes were presented as reasonably good markers of asymptomatic or recently exposed populations [57], but still need to be addressed in blood banks.

In this work, cases were mostly reported from Mexico in the 1970s and 1980s (when this country had a higher malaria incidence) and in the USA. The incidence of TT malaria among people residing in endemic areas is unknown. TT malaria cases misclassification is probably frequent in populations continuously exposed to malaria vectors in the same areas. Furthermore, a proportion of the population in malaria-endemic countries presents asymptomatic parasitaemia, preventing an accurate assessment of whether malaria occurring after transfusion is actually TT malaria [52]. TT malaria surveillance is a challenge in active transmission areas, likely reflected in under-reporting of cases in Central America and Amazon areas. TT malaria can therefore only be distinguished from naturally transmitted cases by genotyping to demonstrate that the parasite in the transfusion recipient is identical to the paired transfused blood unit [58]. A limitation of this approach is that it fails to detect low-level *Plasmodium* clones in the transfused blood.

Transfusion-transmitted malaria prevailed in females requiring blood transfusion after gyneco-obstetrical complications. A significant number of cases were also recorded from the older population, with surgical procedures and complications from neoplasias as the most common transfusion indications. As expected, whole blood and packed RBCs were the most common sources of TT malaria in this study, with 94% of the total cases reported. Platelets were responsible for 6% of the TT malaria cases. Previous reports show that platelets, fresh frozen plasma and leukocytes may infrequently transmit malaria [59–61]. Separate components, such as plasma and platelets, may contain merozoites or some RBCs contamination, and parasites in such components have been shown to be highly sensitive to pathogen reduction measures, such as photochemical treatment and long-wavelength ultraviolet light (UV) [62]. In Ghana, a randomised, controlled, clinical trial has shown the efficacy and safety of a whole blood pathogen reduction using UV and riboflavin (Mirasol System) at preventing TT malaria [63].

Most TT malaria cases were caused by *P. malariae*. Conversely, *P. falciparum* was the predominant species found in the identified donors. This disagreement may be due to the fact that many blood units or donor samples associated with transfusion *P. malariae* malaria tested negative or positive only by serology. *Plasmodium malariae* differs from other *Plasmodium* species by its very low levels of parasitaemia, indolent illness and extended duration of asymptomatic infection [50, 64, 65]. Thus, patients generally come to medical attention when a recrudescence is induced by splenectomy performed for reasons unrelated to malaria [66] or when malaria is transmitted by transfusion, as observed in this study. *Plasmodium vivax* was the second causative agent of TT malaria, mostly related to donors coming from Asia and Latin America, whereas *P. falciparum* was associated with donors who lived or visited African countries.

Clinical severity of TT malaria is very different in endemic and non-endemic countries. In this work, lethality was associated with living in a non-endemic country. Actually, estimates of lethality rates ranged from 11% in the USA [67] to 40% in the United Kingdom (UK) [68] and 100% in France [69]. Although healthy adults living in malaria-endemic areas have some immunity to developing clinical malaria, making them able to harbour low levels of parasites without developing clinical symptoms, the recipients of blood transfusions are predominantly patients with severe co-morbidities who are likely to be immunologically compromised. Malaria diagnosis in non-endemic countries where local doctors are not routinely exposed to the disease constitutes a major challenge and requires trained clinical and laboratory personnel [5]. In the absence of early diagnosis and opportune treatment, TT malaria patients may often evolve to poor outcomes. In addition, about 20% of the TT malaria patients in this study presented long incubation periods (more than 3 months), rendering diagnosis even more difficult due to a more improbable association with transfusion.

*Plasmodium falciparum* TT malaria was associated with higher lethality in comparison with *P. malariae* and *P. vivax*. Complex systemic disorder triggered by host inflammatory responses is present in all types of malaria. However, in falciparum malaria there is prominent adhesion and vascular sequestration, leading more frequently to severe disease and death [70]. In Latin American countries where *P. vivax* predominates, there is an assumption that vivax malaria is more benign and therefore TT malaria is not considered an important burden to public health [19]. Interestingly, in this work, severity and lethality were also observed for *P. malariae* and *P. vivax* malaria. Especially amongst patients with co-morbidities which are characteristic of transfusion recipients, *P. vivax* is associated with severe disease [6, 8].

Complications from neoplasias were associated with a higher odd of lethality from TT malaria in comparison with other transfusion indications. Patients undergoing cancer treatment are commonly under immunosuppressive therapy and frequently need blood transfusion due to anaemia episodes from chemotherapy. Transfusion-transmitted malaria in patients with neoplastic diseases increases the risk of severe clinical picture and fulminant evolution, regardless of the *Plasmodium* species [61, 71–73]. In cancer patients, even those at early stages, several coagulation abnormalities lead to an increased tendency to haemostatic dysfunction [74], directly influencing malaria pathogenesis, including disseminated intravascular coagulation and cerebral malaria [75]. Baseline coagulation disorders triggered by neoplastic disease may play a key role in developing malaria complications. Considering that TT malaria in a patient with cancer (and possibly other severe co-morbidities) is always a life-threatening condition, malaria diagnosis must always be considered in patients with a post-transfusion febrile illness. In these cases, however, it is difficult to define the immediate cause of death to malaria rather than the underlying condition.

## Conclusions

The burden imposed by TT malaria in most countries of Latin America with endemic malaria remains a critical knowledge gap. Without this information, it is impossible to estimate whether pre-transfusion screening within endemic settings is a cost-effective option for prevention of TT malaria. Furthermore, there is no screening method available that is practical, affordable and sensitive enough for use by blood banks in American countries where *P. vivax,* a species associated with relapsing episodes and low-level parasitaemia, contributes greatly to transmission. Most available malaria tests, even those based on DNA amplification, may still have limited sensitivity to detect very low levels of parasites (enough to lead to TT malaria), and there is no evidence-based guidance to indicate which malaria screening methods are effective for use by transfusion services in malaria-endemic countries. It is difficult to estimate the incidence of TT malaria among people residing in endemic areas, especially due to TT malaria cases misclassification in populations continuously exposed to vectors, which may lead to a serious underestimation of TT malaria cases. Lethality associated with TT malaria is not negligible, and correlated with living in non-endemic countries when *P. falciparum* was the causative agent and neoplastic diseases were the transfusion indication. In the current context of malaria transmission in Latin America, uncommon ways of transmission regain attention, such as TT malaria, a potential vehicle for parasite re-introduction in areas moving towards elimination. In the absence of a realistic estimate of TT malaria burden, a possible cost benefit analysis of molecular screening or other more sensitive tests in blood donor candidates compared to TT malaria healthcare-associated costs remains a major gap in the literature (Additional file 1).

## Additional file

**Additional file 1.** Epidemiological and clinical characteristics of transfusion-transmitted malaria cases reported in the American continent: a systematic review database.

## Abbreviations

API: annual parasite index
CI: confidence interval
DNA: deoxyribonucleic acid
IIF: indirect immunofluorescence
LAMP: loop-mediated isothermal amplification
LILACS: Latin America and the Caribbean Health Sciences Literature
MEDLINE: Medical Literature Analysis and Retrieval System Online
MeSH: Medical Subject Heading
NAT: nucleic acid testing
OR: odds ratio
PCR: polymerase chain reaction
RBC: red blood cell
RDT: rapid diagnostic test
TT: transfusion-transmitted
USA: United States of America
WHO: World Health Organization
